# Sustainable treatment of ceramic manufacturing wastewater using combined advanced oxidation and coagulation/precipitation processes with green nano zero-valent iron: multi-metal corrosion monitoring

**DOI:** 10.1038/s41598-026-42824-1

**Published:** 2026-03-26

**Authors:** E. Khamis, D. E. Abd-El-Khalek, M. Hagar, Ahmed S. Mahmoud, T. E. Reyad

**Affiliations:** 1 Chemistry Department, Faculty of Science, Alexandria University, Alexandria, 21321 Egypt; 2 Science & Innovation Center of Excellence, SICE, Egyptian Russian University, Badr, Egypt; 3 National Institute of Oceanography and Fisheries (NIOF), Cairo, Egypt; 4 Institute of Environmental Studies, Arish University, Arish, North Sinai Egypt; 5 Al Ezz Dekheila Steel CO., Alexandria, Egypt

**Keywords:** Sustainable treatment, Ceramic wastewater, Nano zero-valent iron, Advanced oxidation, Metal corrosion ANOVA test, Regression modeling, Chemistry, Engineering, Environmental sciences, Materials science

## Abstract

The ceramic industry generates significant amounts of wastewater, which contains many corrosive ions that pose extensive hazard to the integrity of piping systems, equipment, and machinery, resulting in increased maintenance costs, frequent equipment exchange, and downtimes. To address these issues and achieve the Sustainable Development Goals an advanced treatment process using Fenton Oxidation, coagulation/precipitation, and the addition of green “nano Zero-Valent Iron (nZVI)” was applied to “Ceramica Venezia” wastewater, one of the ceramic factories in Egypt. This study provides a unique multi-metal corrosion benchmarking analysis (Carbon Steel, Stainless Steel, and Copper) specifically examining the impact of green-nZVI advanced treatment on ceramic industrial effluent reuse in comparison with the factory’s initial treatment process and untreated ceramic wastewater. The corrosion rate was evaluated using electrochemical techniques, *EIS,* and *PDP* of different metals in these two different treatment protocols. Moreover, statistical tools, including ANOVA and t-test, were applied to interpret the results. Advanced-treated water (ATW) exhibited an 86% reduction in steel corrosion and a minor improvement in stainless steel corrosion; meanwhile, worsening copper corrosion resistance. Accordingly, different treatment strategies should be studied for achieving better copper protection.

**Supplementary Information:**

The online version contains supplementary material available at 10.1038/s41598-026-42824-1.

## Introduction

Industrial wastewater is regarded as a secondary water resource that offers high economic and financial benefits, including a clean, sustainable, and robust environment, as well as contributing to achieving sustainable development goals (SDGs) and mitigating environmental pollution resulting from rapid urban expansion and strong industrial growth^1–4^.

One of the industries that consumes a lot of water during the production processes is the ceramic manufacturing industry, which is also an essential industry in various aspects, including construction, household supplies, and most industrial applications ^5^. The amount of water that is consumed for the production of one square meter of tiles is approximately 20 L; 60% of them are used up in the production of slurry^6^, and the remainder is consumed in the glazing process, which is the main reason behind choosing this industry that produces a huge amount of wastewater throughout all production steps, starting from slurry, passing through spray drying, glazing, coloring, encasing, and polishing, and ending with the cleaning of production area floors^7^.

The enormous amounts of wastewater generated from the ceramic industry mainly consist of muds, insoluble silicates, ferrites, and corrosive materials such as sulfate (about 100–500 ppm) and chlorides (about 100–700 ppm); zinc, lead, and some heavy metals also exist, as well as chemical oxygen demand (COD) (about 150–1000 ppm) and biological oxygen demand (BOD) (about 50–400 ppm)^8^. The existence of such contaminants, literature values from factory specifications, not only poses environmental challenges but also increases the rate of damage to industrial equipment, piping systems, and storage reservoirs, in addition to increasing maintenance costs, reducing functional effectiveness, and increasing the potential for contamination of the advanced ceramic products^9,10^.

The ceramic industry generates wastewater containing suspended solids, heavy metals, and organic pollutants, requiring effective treatment methods such as coagulation-flocculation, which uses chemicals like alum or ferric chloride to aggregate fine particles for sedimentation^11^; electrocoagulation (*EC*), an advanced process that removes metals and colloidal particles via electrochemical reactions^12^; membrane filtration, including ultrafiltration and reverse osmosis, for high-efficiency separation of contaminants^13^; and biological treatment, which employs microorganisms to degrade organic pollutants^14^. Combining these methods can enhance treatment efficiency, ensuring compliance with environmental regulations.

Therefore, in order to properly recycle ceramic wastewater for industrial and agricultural applications, improved treatment, including corrosion inhibitors, should be implemented. Methods that employ environmentally friendly corrosion inhibitors and nanomaterials show promise as substitutes for managing metal corrosion and improving the total quality of recovered wastewater^15,16^. Applying this practice will lessen the corrosive effects, and the water will be fit for reuse in several industrial and agricultural uses. Although they are necessary, conventional treatment techniques, including filtration, sedimentation, and chemical treatment, have several drawbacks and usually do not provide enough corrosion resistance^17–20^.

This study aims to achieve the following goals: firstly, to investigate the effect of untreated ceramic wastewater generated from the "Ceramica Venezia factory" compared to two treatment processes: the factory’s initial treatment by using coagulation/precipitation process (Alum) and an advanced treatment using an advanced oxidation process (Fenton Oxidation) followed by coagulation /precipitation process (Ferric Chloride) and addition of green “nano Zero-Valent Iron (nZVI) on the corrosivity of commonly used metals. In this approach, the corrosivity of mild steel, stainless steel, and copper were measured in various wastewater treatment conditions using electrochemical impedance spectroscopy (*EIS*) and Potentiodynamic polarization (*PDP*). Secondly, to evaluate how well the advanced treatment in ceramic wastewater helps to mitigate metal corrosion rates, and finally, to ascertain whether the differences in corrosivity among different treatment processes are statistically significant or not by applying statistical tools such as one-way ANOVA and t-test as well as applying the machine-learning modeling for predicting the effect of medium parameters on steel, stainless-steel and copper corrosion resistance. By achieving these objectives, this research will provide valuable insights into ceramic wastewater treatment approaches and their impact on the corrosion of different commonly used metals. Additionally, it will explore the potential of utilizing recycled wastewater in specific applications, such as agriculture and certain industrial uses.

## Results and discussion

### Assessment of corrosion behavior of mild steel in different ceramic wastewater treatments

#### Electro-impedance spectroscopy measurements (EIS)

**Fig. 1:**
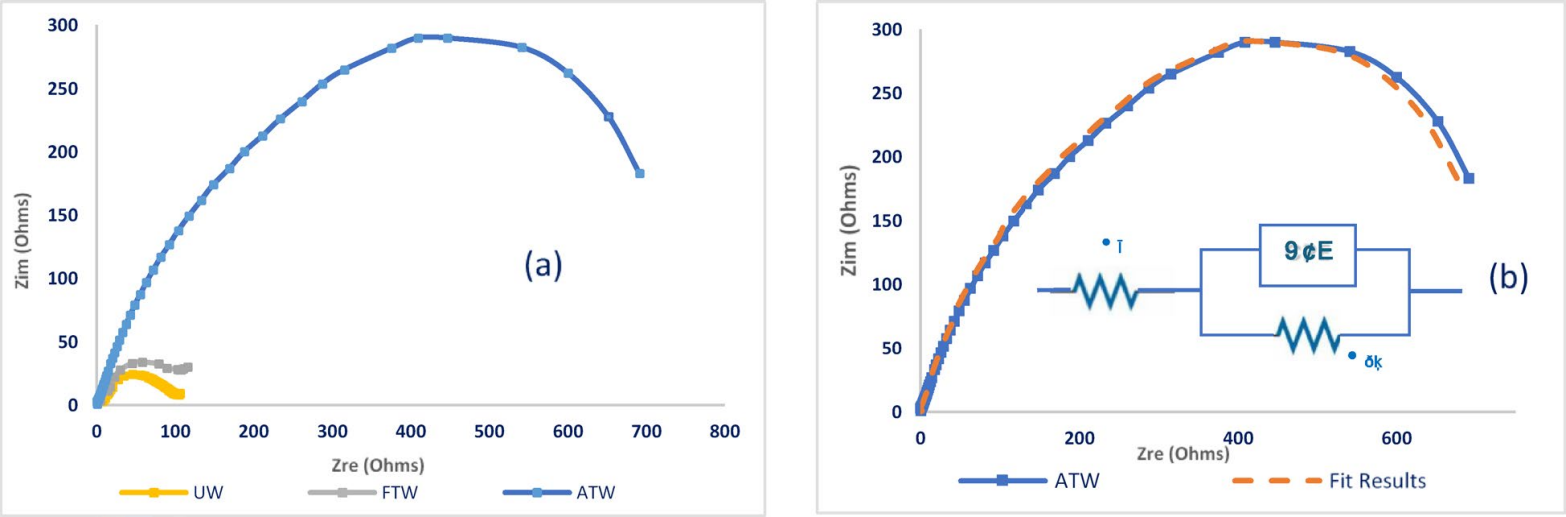
**(a)** Nyquist plots for steel (n = 3, 30 °C); ATW shows the largest semicircle (*R*_ct_ = 738 Ω·cm^2^). **(b)** Fitting of the ATW Nyquist plot using the equivalent circuit model.

The Nyquist plots for steel in untreated (UW), factory-treated (FTW), and advanced-treated wastewater (ATW) samples, respectively, were studied as shown in Fig. 1a. The impedance response exhibited a single distorted loop of capacitive type, which suggests that the corrosion mechanism is driven by a process of charge transfer^21^. It is also noted that these semicircles’ size is increased in advanced-treated water (ATW) compared to factory-treated water, which reflects that ATW exhibits enhanced corrosion resistance for steel than the factory-treated water (FTW).

The percentage of corrosion resistance (*CR%*) was determined by utilizing the following Eq. ^22^.

1 $$CR\% = \left[ {1 - \frac{{R_{ct}^{o} }}{{R_{ct} }}} \right]x{ }100$$

where *R*_ct_ and *R*_ct_^o^ are the resistance to charge-transfer for treated and untreated wastewater, respectively.

In this type of Nyquist diagram, Randles’ circuit without a Warburg diffusion element^23^ can be used to model it by modifying this circuit through substituting the Constant Phase Element (*CPE*) for capacity, which enables us to account for the capacitor’s imperfect structure, the capacity of the electric double layer (*EDL*) can be represented by the module *Q* of the *CPE* element with a phase factor n near 1 where the more diffuse and/or inhomogeneous the structure’s near-electrode layer, the lower the phase factor *n*. The impedance spectra from various Nyquist plots were studied by fitting the experimental data to the simple equivalent circuit model (shown in the Fig. 1b), which consists of the solution’s resistance (*R*_s_) and *CPE*, which is assembled in parallel to the charge transfer resistance (*R*_ct_) in the circuit. The *CPE* is defined by two parameters, *Q*, and n. The impedance, *Z*, of the *CPE* is expressed as^24–26^:

2 $$Z_{{{\mathrm{CPE}}}} = Q^{ - 1} \left( {i\omega } \right)^{ - n}$$

where, *i* = (− 1)^1/2^, *ω* is a frequency in rad/s, *ω* $=2\pi f$ and f is the frequency in Hertz. If n equals one, then Eq. (2) is equivalent to that of a capacitor, *Z*_C_ = (*iωC*)^-1^, where *C* is the ideal capacitance for a nonhomogeneous system, n-value ranges 0.9–1^27^. The computer-fitting data is summarized in Table 1.

**Table 1:** Results of computer-fitting for the steel impedance spectra in untreated (UW), factory-treated (FTW), and advanced-treated ceramic wastewater (ATW).

Solution	*R*_s,_ Ω.cm^2^	*Q, µF.cm*^-1^	*n*	*R*_ct_, Ω.cm^2^	ꭓ^2^	*CR%*
UW	15.51 ± 0.39	748 ± 0.49	0.56 ± 0.016	100.1 ± 5.1	9.5 × 10^–4^	–
FTW	13.79 ± 0.54	635 ± 0.35	0.60 ± 0.027	144.2 ± 7.2	4.3 × 10^–4^	30 ± 1.5%
ATW	4.61 ± 0.21	135 ± 0.12	0.71 ± 0.003	738.3 ± 15.5	4.0 × 10^–4^	86 ± 2.1%

The data in Table 1 illustrates that the *R*_*s*_ is increasing from UW, FTW to ATW, which may be due to the ionic conductivity decrease by the perception of a protective layer^28,29^.

In the same direction, the double layer capacitance *Q* is decreasing with increasing the charge transfer resistance *R*_ct*,*_ which is attributed to protective layer formation on the metal/solution interface with its thickness increases and becomes more compact, and less permeable^30,31^, which interprets the decrease of mild steel corrosion tendency in the following order *UW* > *FTW* > *ATW*.

### Potentiodynamic polarization measurements (PDP)

**Fig. 2:**
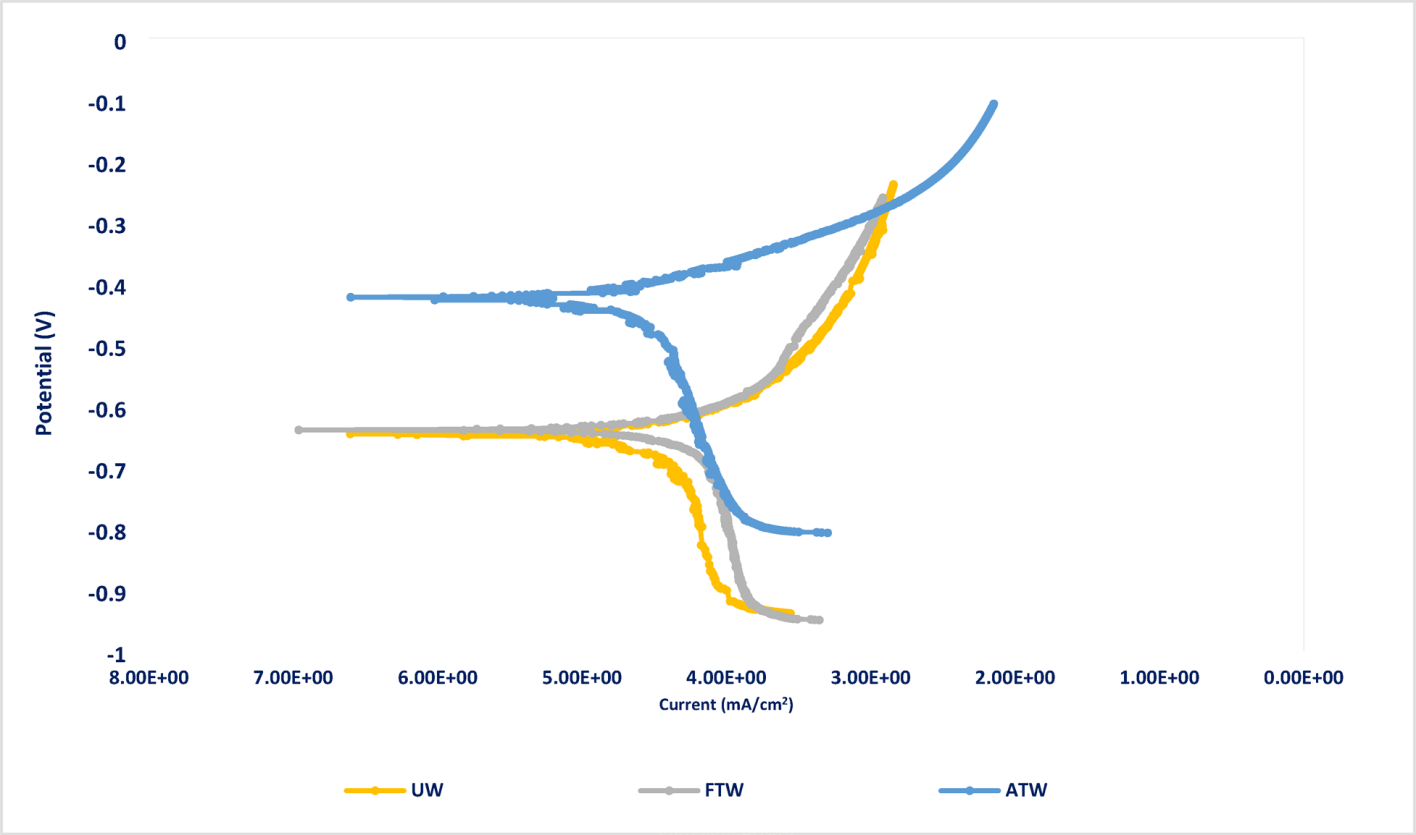
Potentiodynamic polarization curves for steel (n = 3, 30 °C); in untreated wastewater (UW), factory-treated water (FTW), and advanced-treated water (ATW), where ***E***_corr_ substantially shifted to a more noble value of -430 mV rather than -659 mV in UW.

The Potentiodynamic Polarization measurements for steel in the three wastewater samples are shown in Fig. 2. The electrochemical parameters of mild steel performance in UW, FTW, and ATW samples, such as corrosion potential (*E*_corr_), current density of corrosion (*i*_corr_), Tafel slopes (*β*_c_ and *β*_a_), and protection efficiency (*%P*), are given in Table 2. The current density of corrosion was estimated through the intersection of Tafel’s anodic and cathodic lines, and the percentage of protection efficiency (*%P*) was determined from polarization measurements using Eq. (3) ^32,33^.

3 $$\% P = [\left( {i_{{{\mathrm{corr}}}} )_{o - } \left( {i_{{{\mathrm{corr}}}} } \right)} \right]/(i_{{{\mathrm{corr}}}} )_{o }$$

where (*i*_corr_)_o_ and (*i*_corr_) represent the corrosion current density in the untreated and treated wastewater, respectively.

**Table 2:** The electrochemical polarization parameters of steel in untreated (UW), factory-treated (FTW), and advanced-treated ceramic wastewater (ATW).

Solution	-*E*_corr_				
(mV *vs. SCE*)	*β*_a_ (mV)	-*β*_c_ (mV)	*i*_corr_ mA/cm^2^	*%P*	
UW	− 659	34.95	55.78	10.01	–
FTW	− 638	63.25	45.93	7.11	29 ± 1.9%
ATW	− 430	68.60	79.80	1.81	82 ± 2.7%

Results in Table 1 display a remarkable decrease in the corrosion current density and an increase in protection efficiency in the advanced-treated sample (ATW) compared to the factory-treated sample (FTW) and the untreated wastewater (UW), which is enhanced from 29% in FTW to 82% in ATW. These results align with the data obtained from electrochemical impedance spectroscopy (*EIS*).

Moreover, the corrosion potential (*E*_corr_) was significantly affected by the advanced treatment process and shifted to a more positive value from − 659 mV (FTW) to − 430 mV (ATW). This substantial shift of 229 mV indicates that the advanced treatment of ceramic wastewater significantly reduces the thermodynamic driving force of steel corrosion, making the steel less disposed to corrosion^37^. The advanced-treated water (ATW) shifts both cathodic and anodic portions of polarization curves to more noble values compared to UW, where the Tafel anodic slope, *β*_a*,*_ is shifted from 35 mV in *UW* to 69 mV in *ATW,* and the Tafel cathodic slope, *β*_c_, is shifted from 56 mV in *UW* to 80 mV in *ATW,* which means blocking of both anodic and cathodic sites, and ATW acts as a mixed-type inhibitor^34–36^. The reason behind the enhanced corrosion resistance of steel in ATW may be attributed to addition of nZVI where, it acts as a superior sacrificial reductant, highly active “oxygen scavenger” because of its extremely small particle size (1–100 nm) and a massive surface-area-to-volume ratio^37^ so, the nZVI reacts rapidly with the dissolved oxygen, consuming it before it reaches to the metal surface^38^ and generates reactive oxygen species Fe^2+^ions undergoing the following Eq. (4):

4 $$Fe^{0} \left( {{\mathrm{nZVI}}} \right) \, + O_{2} + H_{2} O \to Fe^{2 + } + 2OH^{ - }$$

By removing the dissolved oxygen, the nZVI effectively stifles the cathodic (reduction) on the steel surface. Moreover, the rapid oxidation of nZVI produces a high-quality iron oxides Fe (II)/Fe (III) mixture, mainly magnetite according to Eq. (5), which forms precipitate onto the metal surface, forming a robust, non-porous physical barrier that subsequently suppresses both anodic dissolution and residual cathodic activity reaction^39,40^.

5 $$6Fe\left( {II} \right) \, + \, O_{{}} + \, 6 H_{2} O \, \to 2 \, Fe_{3} O_{4} + \, 12H^{ + }$$

This mechanism is further supported by the alkaline *pH* shift in ATW *(pH* = *7.2)* than FTW *(pH* = *4.1)* as observed in Table 10, due to the generation of *OH*^*-*^ ions during the initial oxygen scavenging process (Eq. 3) that also facilitates the precipitation of the stable *Fe*_*3*_*O*_*4*_ (magnetite) layer.

### Assessment of the corrosion behavior of stainless steel in different ceramic wastewater treatments

#### Electro-impedance spectroscopy measurements (EIS)

**Fig. 3:**
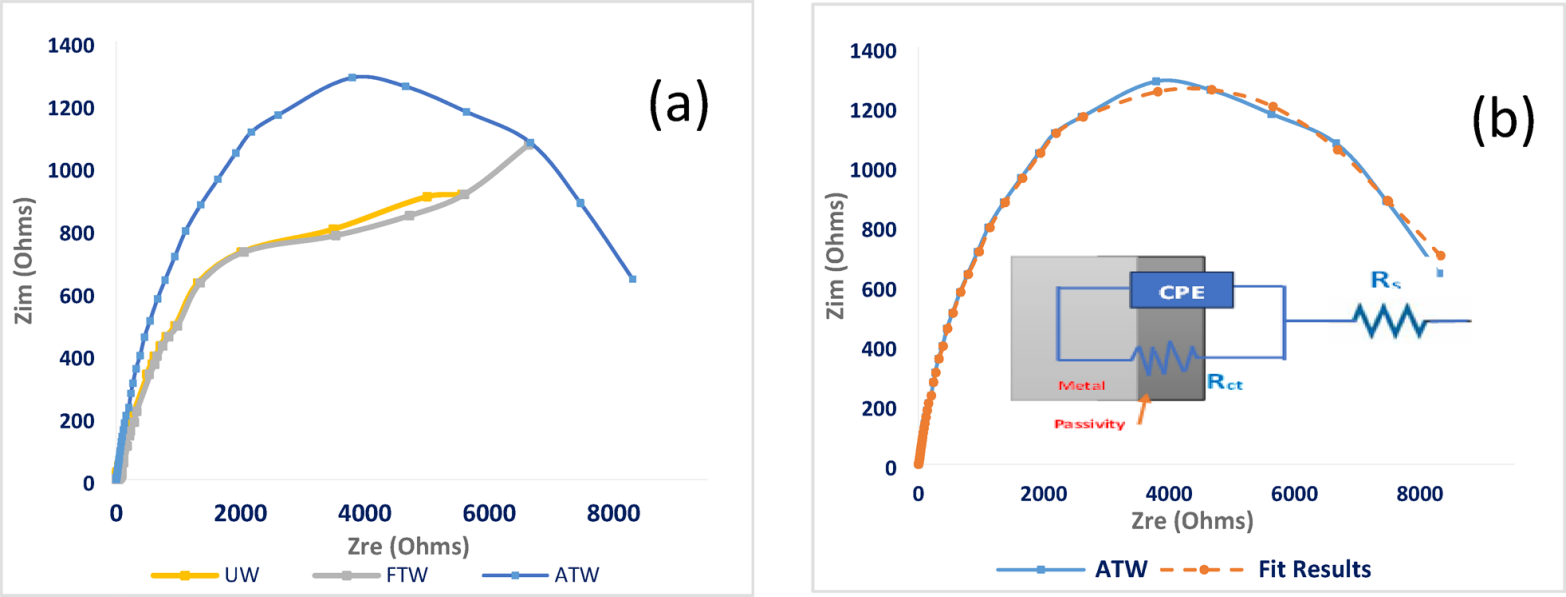
**(a)** Nyquist plots for stainless steel (n = 3, 30 °C); ATW shows the largest semicircle (*R*_ct_ = 8800 Ω·cm^2^). **(b)** Fitting of the ATW Nyquist plot using the equivalent circuit model.

The Nyquist plots for stainless steel in untreated (UW), factory-treated (FTW), and advanced-treated wastewater (ATW) samples, respectively, were studied as shown in Fig. 3a. The impedance response exhibited distorted semicircles, with the size of the semicircles slightly increased in the advanced-treated samples. Meanwhile, the factory-treated sample exhibited lower corrosion resistance than the untreated wastewater. Consequently, the advanced treated sample’s.

#### Corrosion resistance showed a small improvement^41^.

The electro-impedance spectra obtained from various Nyquist plots were studied through fitting the experimental data to the simple equivalent circuit model that is mentioned in Fig. 3b, as it was reported that, the Nyquist plots of stainless steel at lower temperatures, Randle-like features are observed, which are attributed to the passive film existing on the surface of the metal^42^.

**Fig. 4:**
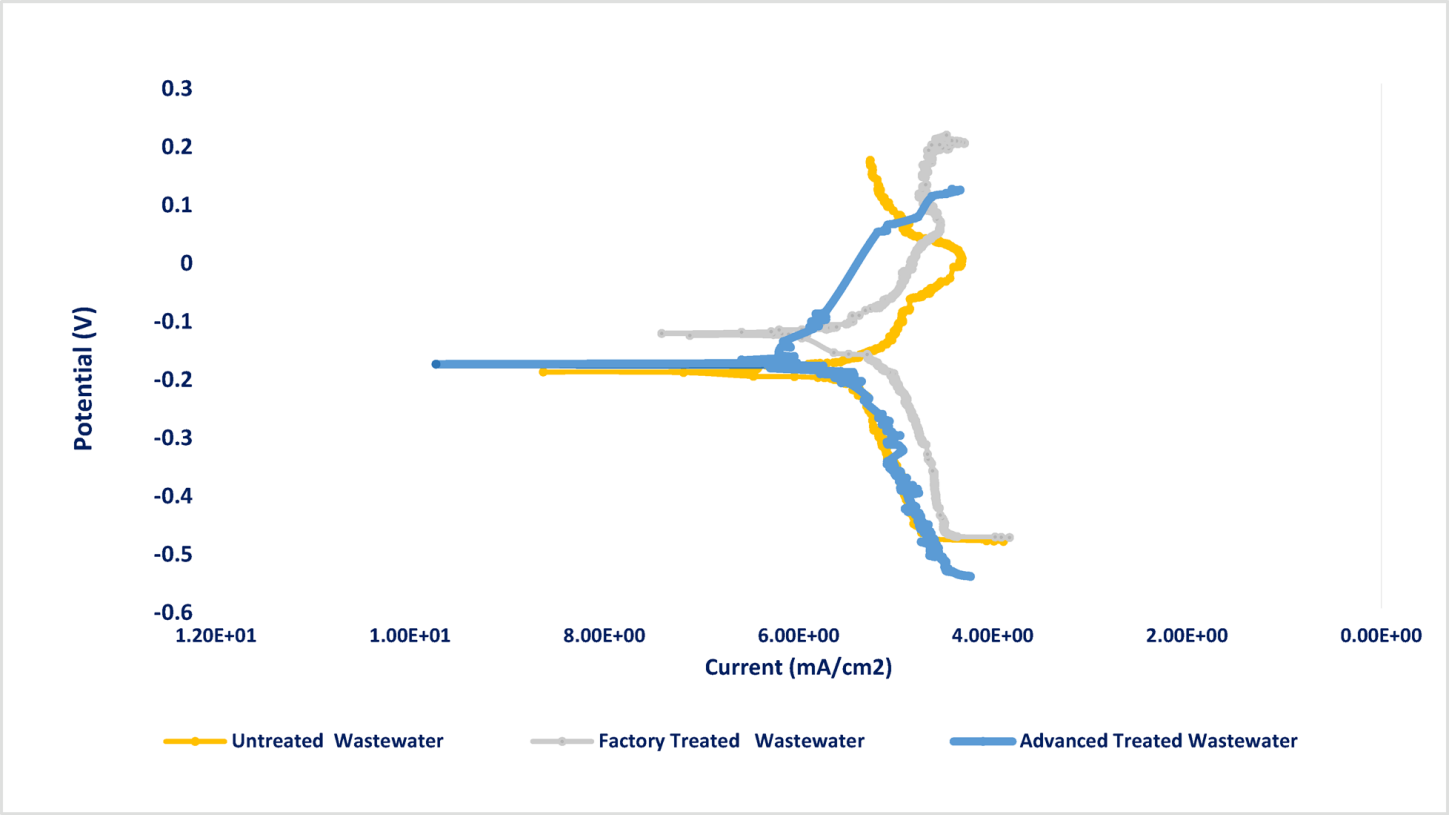
Potentiodynamic polarization curves for stainless-steel (n = 3, 30 °C); in untreated wastewater (UW), factory-treated water (FTW), and advanced-treated water (ATW), where ***E***_corr_ slightly shifted to a more noble value of -120 mV than -193 mV in UW.

The data in Table 3 illustrates that the passive film resistance (*R*_ct_) slightly decreases in factory-treated water (FTW) compared to untreated water (UW), which means that the medium becomes more corrosive for stainless steel in the case of *FTW* than *UW*. This decrease in the passive film *R*_ct_ is due to the metal surface becoming more prone to local attack; meanwhile, the advanced-treated water (ATW) exhibits fewer corrosive properties for stainless steel with higher charge transfer resistance^43^. The factory’s existing wastewater treatment relies on aluminum sulfate coagulation, which partially removes organic contaminants but leaves residual pollutants untreated. Additionally, shifting the *pH* value to a more acidic value of 4.1 also helps in destabilizing the passivation layer. In contrast, the advanced oxidation process (*AOP*) effectively degrades persistent organic contaminants, enabling ferric chloride coagulation to enhance contaminant removal through precipitation, applying the nano Zero Valent Iron “nZVI” that possesses anticorrosion functions^44^. Furthermore, the higher pH of ATW “7.2” helps in stabilizing the formed passive layer on the metal surface. This observation is clearly supported by increasing pseudo capacitance of passive film (*Q*) in *FTW (pH* = *4.1)*, which indicates a decrease in the thickness of the passive film, in contrast to *ATW (pH* = *7.2),* which shows a decrease in capacitance and accordingly an increase in passive film thickness^45^. The corrosivity of stainless steel increased by 18% in factory-treated water, whereas it was slightly inhibited by 9% in the advanced treated wastewater samples (Fig. 4).

**Table 3:** Results of computer-fitting for the stainless-steel impedance spectra in untreated (UW), factory-treated (FTW), and advanced-treated ceramic wastewater (ATW).

Solution	*R*_s_, Ω.cm^2^	*Q, µF.cm*^*-1*^	*n*	*R*_ct_, Ω.cm^2^	ꭓ^2^	*CR%*
UW	95.21 ± 0.61	36.67 ± 0.46	0.76 ± 0.03	7969 ± 65	5.5 × 10^–3^	–
FTW	81.01 ± 0.43	42.05 ± 0.38	0.74 ± 0.02	6740 ± 59	6.3 × 10^–3^	–
ATW	97.81 ± 0.71	26.38 ± 0.45	0.85 ± 0.04	8800 ± 67	1.0 × 10^–4^	9.4 ± 0.4%

### Potentiodynamic polarization measurements (PDP)

The Potentiodynamic polarization measurements for stainless-steel in untreated (UW), factory-treated (FTW), and advanced treated wastewater (ATW) samples, respectively, were studied as shown in Fig. 6. are given in Table 4.

**Table 4:** The electrochemical polarization parameters of stainless steel in untreated (UW), factory-treated (FTW), and advanced-treated ceramic wastewater (ATW).

Solution	-*E*_corr_				
(mV vs. *SCE*)	*β*_a_ (mV)	-*β*_c_ (mV)	*i*_corr_ mA/cm^2^	*%P*	
UW	-193	19	30	0.94	–
FTW	-181	45	60	1.10	–
ATW	-120	81	37	0.87	7.5 ± 0.2%

From the obtained data, the current density of corrosion is slightly increased from 0.94 mA/cm^2^ in untreated wastewater (UW) to 1.1 mA/cm^2^ in factory-treated water (FTW), associated with a slight change in corrosion potential (*E*_corr_), which indicates that *FTW* promotes more corrosion of stainless steel than the untreated wastewater medium. Furthermore, the corrosion potential (*E*_corr_) is significantly influenced by the advanced treatment process, where the corrosion potential is shifted to a more positive noble value of 73 mV, from − 193 mV in UW to − 120 mV in *ATW.* This suggests a retardation of the corrosion thermodynamic driving force, and the metal surface becomes less prone to electrochemical attack. The development of an adsorbed layer on the SS surface, which leads to a slight enhancement in protection efficiency of 7.5%, was observed.

Additionally, the polarization behavior indicates that *ATW* affects both anodic and cathodic reactions. Increasing Tafel slopes for *ATW* (*β*_a_ = 81 mV and *β*_c_ = 37 mV) suggests that the treatment suppresses both metal dissolution and reduction reactions, and *ATW* acts as a mixed-type inhibitor, blocking active sites for both cathodic and anodic processes^46–48^.

### Assessment of corrosion behavior of copper in different ceramic wastewater treatments

#### Electro-impedance spectroscopy measurements (EIS)

**Fig. 5:**
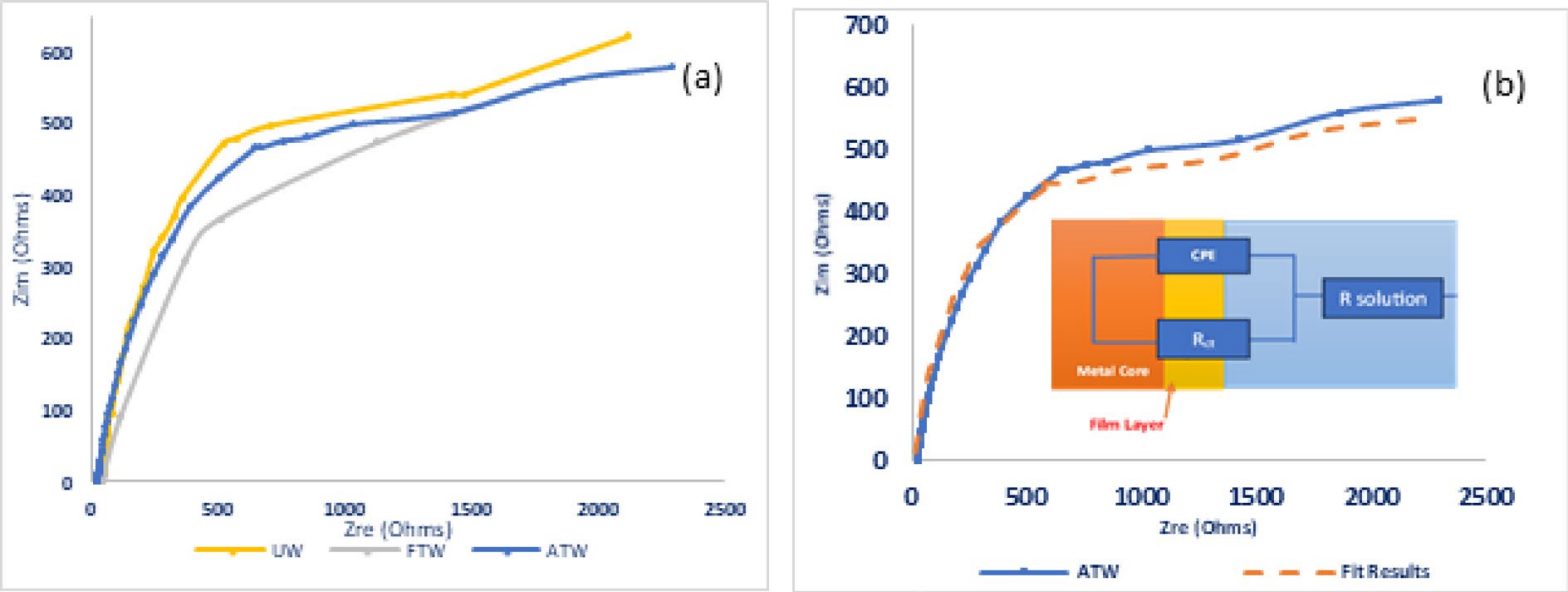
**(a)** Nyquist plots for copper (n = 3, 30 °C); ATW shows a moderate semicircle (*R*_ct_ = 1575 Ω·cm^2^). **(b)** Fitting of the ATW Nyquist plot using the equivalent circuit model.

The Nyquist plots for copper dissolution in untreated (UW), factory-treated (FTW), and advanced-treated wastewater (ATW) samples, respectively, were studied as shown in Fig. 5a.

The time-constant charge transfer and *EDL* capacitance are responsible for the observed semi-circles at high frequencies, which are generally linked to the relaxation of electrical double-layer capacitors and whose diameters indicate the charge-transfer resistances.

The impedance response exhibited a depressed capacitive semicircle, demonstrating that corrosion of copper occurs under charge transfer control^49–51^. The size of the semicircles is slightly depressed on goingfrom untreated wastewater (UW) to factory-treated water (FTW) to advanced-treated water (ATW), which illustrates that both treatment methods have become more corrosive for copper. The observed effect may be attributed to two primary factors: (1) organic contaminants like grease could isolate the metal from its ions in solution by forming a protective barrier^52,53^, (2) elevated sulfate ions from factory-treated wastewater (FTW) and with chloride ions from advanced-treated wastewater (ATW) likely promote copper corrosion^54–56^ (3) in addition to the presence of higher total phosphorus content in *UW* than *FTW* than *ATW* where, EPA says you need 0.33–1.0 mg/L phosphorus for protection. ATW has 0.52 mg/L total *P*, but if only half is active phosphate (~ 0.26 mg/L), it’s probably too low for good films, accordingly, decreases the active copper surface area and soluble Cu^2+^ release by forming low-solubility copper-phosphate surface films, such as *Cu₃(PO₄)₂* or mixed Cu-phosphate layers these mechanisms collectively explain the reduced metal reactivity and increased corrosion resistance observed in the UW^57,58^.

The electro-impedance spectra obtained from various Nyquist plots were analyzed by fitting the experimental data to the constant-phase element (*CPE*) circuit model, which is mentioned in Fig. 5b. The results of the computer fitting for the impedance spectrum are presented in Table 5^59–61^ The data in Table 5 illustrates that the film resistance (*R*_ct_) is slightly decreased when going from untreated wastewater (UW) to factory-treated water (FTW) advanced-treated water (ATW). As seen, the dissolution of copper is increased in factory-treated wastewater by 11% and in advanced treated wastewater by 4% to the untreated wastewater. The data is also supported by the increasing pseudo capacitance of the film layer (*Q*) in *FTW* than *ATW* than *UW*, which indicates a decrease in the thickness of the protection film on the copper surface in both treated water media^62^.

**Table 5:** Computer fit results of the impedance spectra for copper in untreated (UW), factory-treated (FTW), and advanced -treated ceramic wastewater (ATW).

Solution	*R*_s_, Ω.cm^2^	*Q, µF.cm*^*-1*^	*n*	*R*_ct_, Ω.cm^2^	ꭓ^2^	*CR%*
UW	42.13 ± 0.41	16.3 ± 0.87	0.81 ± 0.01	1641 ± 17	3.8 × 10^–4^	–
FTW	49.01 ± 0.51	48.60 ± 0.45	0.76 ± 0.04	1480 ± 14	3.9 × 10^–3^	–
ATW	25.20 ± 0.27	31.30 ± 0.38	0.74 ± 0.02	1575 ± 16	5.0 × 10^–3^	–

### Potentiodynamic polarization measurements (PDP)

**Fig. 6:**
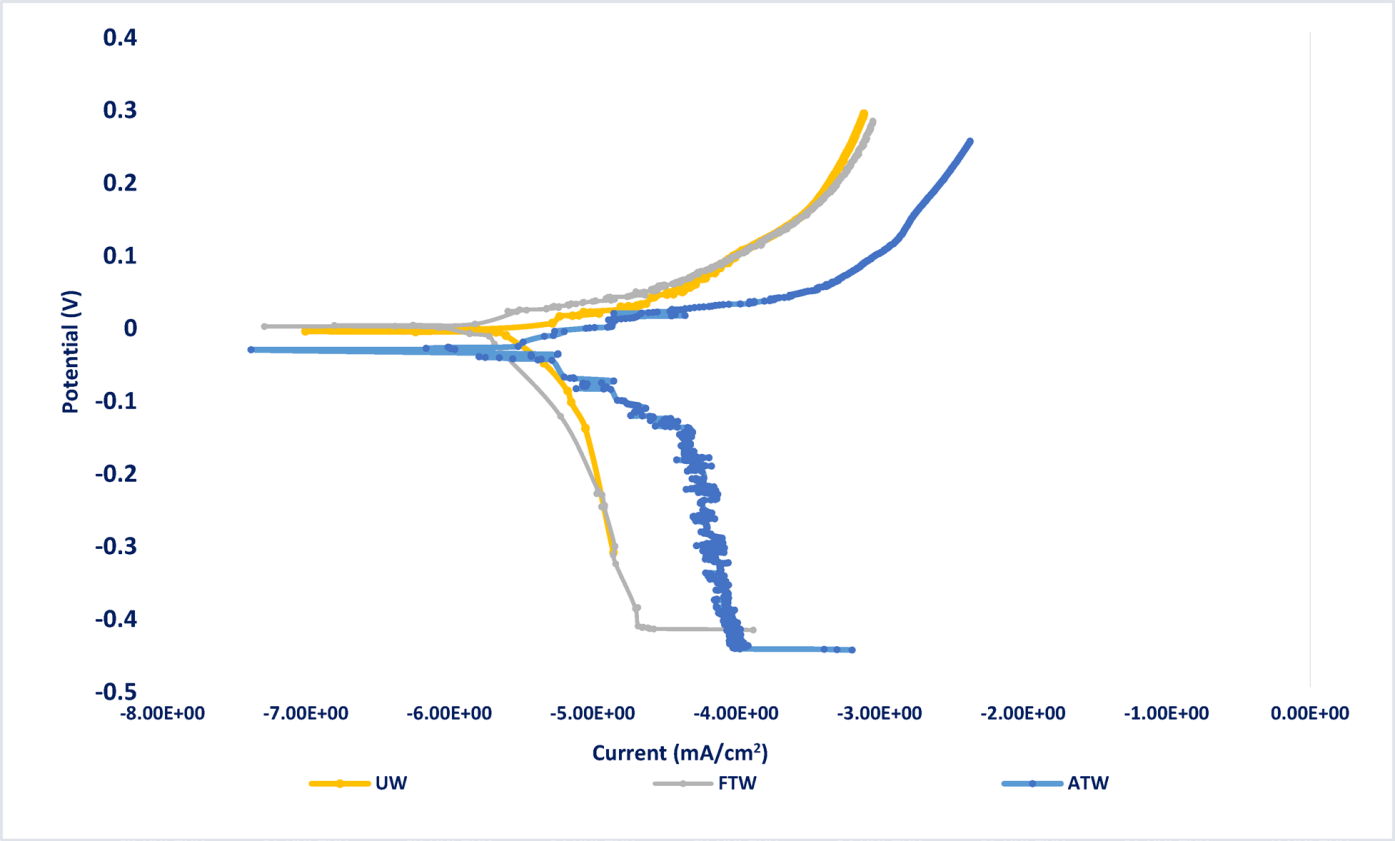
Potentiodynamic polarization curves for copper (n = 3, 30 °C); in untreated wastewater (UW), factory-treated water (FTW), and advanced -treated water (ATW), where ***E***_corr was_ worse shifted to a more negative value of − 33 mV rather than − 29 mV in UW.

The Potentiodynamic polarization measurements for copper in untreated (UW), factory-treated (FTW), and advanced treated wastewater (ATW) samples, respectively, were studied as shown in Fig. 6. The electrochemical parameters of copper performance in *UW*, *FTW*, and *ATW* samples, such as the corrosion potential (*E*_corr_), the current density of corrosion (*i*_corr_), Tafel slopes (*β*_a_ and *β*_c_), and protection efficiency (*%P*), are given in Table 6.

**Table 6:** The electrochemical polarization parameters of copper in untreated (UW), factory-treated (FTW), and advanced -treated ceramic wastewater (ATW).

Solution	-*E*_corr_ (mV vs. *SCE*)	*β*_a_ (mV)	-*β*_c_ (mV)	*i*_corr_ mA/cm^2^	*%P*
UW	− 29	66	104	1.21	–
FTW	− 24	80	94	1.33	–
ATW	− 33	67	180	1.27	–

The current density of corrosion is slightly increased from 1.21 mA/cm^2^ in untreated wastewater (UW) to 1.27 mA/cm^2^ and 1.33 mA/cm^2^ in advanced-treated water (ATW) and factory-treated water (FTW), respectively. These results are associated with a minor change in corrosion potential (*E*_*corr*_), which indicates that *FTW* becomes more corrosive for copper than *ATW* and *UW,* with a decrease in corrosion protection percentage of 10% and 5%, respectively. These findings are consistent with the data obtained from EIS, indicating that both treatment processes promote copper dissolution more than untreated wastewater ^63^.

### Statistical analysis of ceramic wastewater treatment efficiencies

As the steel showed a good performance in different ceramic wastewater treatment media, the statistical comparison of *CR% (*Corrosion Resistance) of steel performance in Factory Treated Water (FTW) and Advanced-treated water (ATW) was analyzed using both one-way ANOVA “Tukey’s HSD” and t-test at a 95% Confidence Interval (*CI*) applying the Minitab program “Version 20.4”^64^. In the same way, the effect of *ATW* on the ***corrosion resistance*** of different metals.

### Study the effect of different treatments on Steel corrosion

Table (7) illustrates the corrosivity of steel in untreated wastewater (UW), factory-treated water (FTW), and advanced -treated water (ATW) at a confidence level of 95%; Table (8) illustrates the analysis of variance (ANOVA) for corrosion resistance of steel in factory-treated water (FTW) and advanced -treated water (ATW), respectively.

From the data in Tables 7 and 8, the comparison between *Untreated Wastewater* and *factory-treated wastewater* resulted in a *t-*value of 29.26 and a *p-*value of 0.00018**.** Since ***p* < *0.05***, the difference is statistically significant, which indicates that factory treatment significantly improves the *CR%* compared to untreated wastewater, with a mean resistance improvement of 30%.

**Table 7:** The corrosivity of untreated wastewater (UW), factory-treated water (FTW), and advanced -treated water (ATW) at a confidence level of 95% (*n* = *3*, *normality of data confirmed *via* the Ryan-Joiner test at ****p* > *0.05****)*.

Treatment strategy	Mean CR%	StDev	95% CI (Low)	95% CI (High)
UW	–	–	–	–
FTW	29.73%	1.75%	27.45%	32.02%
ATW	86.60%	0.22%	84.31%	88.88%

**Table 8:** The analysis of variance (ANOVA) and t-test data for corrosivity factory-treated water (FTW), and advanced -treated water (ATW). (*n* = *3*, *normality of data confirmed *via* the Ryan-Joiner test at p* > *0.05)*.

Comparison	Diff. of means (%)	SE of Diff	*t*-value	*P*-value	Interpretation
UW vs. FTW	29.73	1.02	29.26	0.00018	Significant (p < 0.05)
UW vs. ATW	86.59	0.99	85.21	0.000014	Significant (p < 0.05)
FTW vs. ATW	56.86	1.01	55.95	0.000032	Significant (p < 0.05)

Moreover*,* the comparison between *untreated* and *advanced treated wastewater* resulted in a *t-*value of 85.21 and a *p-*value of 0.000014. This illustrates an extremely significant improvement in *CR%* due to the advanced treatment process, which exhibits efficient performance in corrosion resistance with a mean enhancement of 86.59%. That is also higher than *factory-treated* water by 56.86% which is statistically significant (*p* = *0.000032*) and highlights the necessity of the advanced treatment step for maximum efficiency.

### Multi-metal corrosion evaluation post-nZVI “ATW Medium”.

A statistical study was performed to compare the corrosion ***evaluation*** of the three different metals: Steel, Stainless Steel, and Copper, due to the effect of the advanced-treated water (ATW), at a confidence level of 95%.

The results in Table 9 illustrate that there is a statistically significant difference between the corrosion resistance of Steel and Stainless Steel in *ATW* with *t*-value (160.76) and *p*-value (0.00001). Also, Steel demonstrates vastly superior corrosion resistance compared to copper with a difference of means (90.36%) and *p*-value (0.00001).

**Table 9:** The analysis of variance (ANOVA) and *t-test* data for corrosion resistance of Steel, Stainless-Steel, and Copper in advanced -treated water (ATW). (*n* = *3*, *normality of data confirmed *via* the Ryan-Joiner test at ****p* > *0.05****)*.

Comparison	Diff. of means (%)	SE of Diff (%)	*t*-value	*P*-value	Interpretation
Steel vs. Stainless Steel	77.07	0.45	160.76	0.00001	Significant (p < 0.05)
Steel vs. Copper	90.36	0.34	188.49	0.0001	Significant (p < 0.05)
Stainless Steel vs. Copper	13.29	0.13	27.73	0.0022	Significant (p < 0.05)

**Fig. 7:**
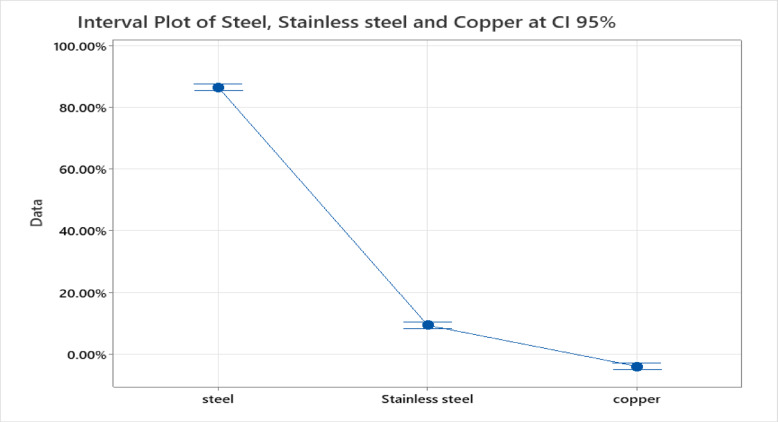
The interval plot of the mean *CR%* for steel, stainless steel, and copper in advanced-treated water (ATW) at a Confidence Interval of 95% (Dataset Size is limited; n = 9).

From the statistical data, it is observed that Steel has the highest corrosion resistance of 86.6% and indicates superior corrosion resistance in advanced-treated water (ATW), Stainless Steel exhibits low corrosion resistance of 9.34%, but is significantly lower than Steel. Meanwhile, Copper shows negative corrosion resistance, meaning it is more susceptible to dissolution under the tested conditions. From the statistical analysis data, Fig. 7 illustrates the interval plot of the mean corrosion resistivity for steel, stainless steel, and copper in advanced-treated water *ATW* at a Confidence Interval of 95%. The Figure displays the higher corrosion resistance of steel than stainless steel in ATW, whereas copper becomes more corrosive at *ATW* conditions.

### Linear regression modeling for corrosion resistance prediction

Linear Regression models were employed to predict steel corrosion resistance (Response) as a function of medium parameters such as *pH*, *TDS*, and total Phosphates (*TP*). Among different algorithms, the linear regression model was deployed and demonstrated the highest predictive performance (*R*^*2*^ = *0.99*, impressive but *n* = *3* risks overfitting) for three wastewater conditions (UW, FTW, ATW) with measured *pH*, *TDS*, and total phosphorus serving as predictors^65,66^.

The developed mathematical model for the corrosion behavior of mild steel, stainless steel, and copper in the various ceramic wastewater treatment environments can be expressed by the following equations: the values mentioned between parentheses represent the coefficients intervals at *CI 95%*:

### Steel corrosion resistance modeling equation

6 $$CR\% = \, 110.74 \, \left( { \pm 6.50} \right) - 31.179 \, \left( { \pm 1.62} \right)pH + \, 0.035734 \, \left( { \pm 0.0012} \right)TDS\left( {ppm} \right)$$

Equation (6) can be used to forecast the corrosion resistance of the mild steel in the studied conditions. The constant in the equation is the residue.

### Stainless steel corrosion resistance modeling equation

7 $$CR\% = - {79}.{96 }\left( { \pm {2}.{41}} \right) \, + { 17}.{37}0 \, \left( { \pm 0.{69}} \right)pH - \, 0.00{6386 }\left( { \pm 0.000{5}} \right)TDS\left( {ppm} \right)$$

Equation (7) can be used to forecast the corrosion resistance of stainless steel in the studied conditions. The constant in the equation is the residue.

### Copper corrosion resistance modeling equation

8 $$\begin{aligned} CR\% = & \, - { 3}.{6341}\left( { \pm 0.{18}} \right) \, - { 3}.{7}0{72 }\left( { \pm 0.{2}0} \right)pH + \, 0.00{37 }\left( { \pm 0.000{2}} \right)TDS\left( {ppm} \right) \\ + { 1}0.{3135 }\left( { \pm 0.{61}} \right)TP\left( {ppm} \right) \\ \end{aligned}$$

Equation (8) can be used to forecast the corrosion resistance of the copper in the conditions studied. The constant in the equation is the residue. In this model, the effect of Total phosphorus could not be neglected as it influences enhancing the corrosion resistance of copper, as illustrated before.

**Fig. 8:**
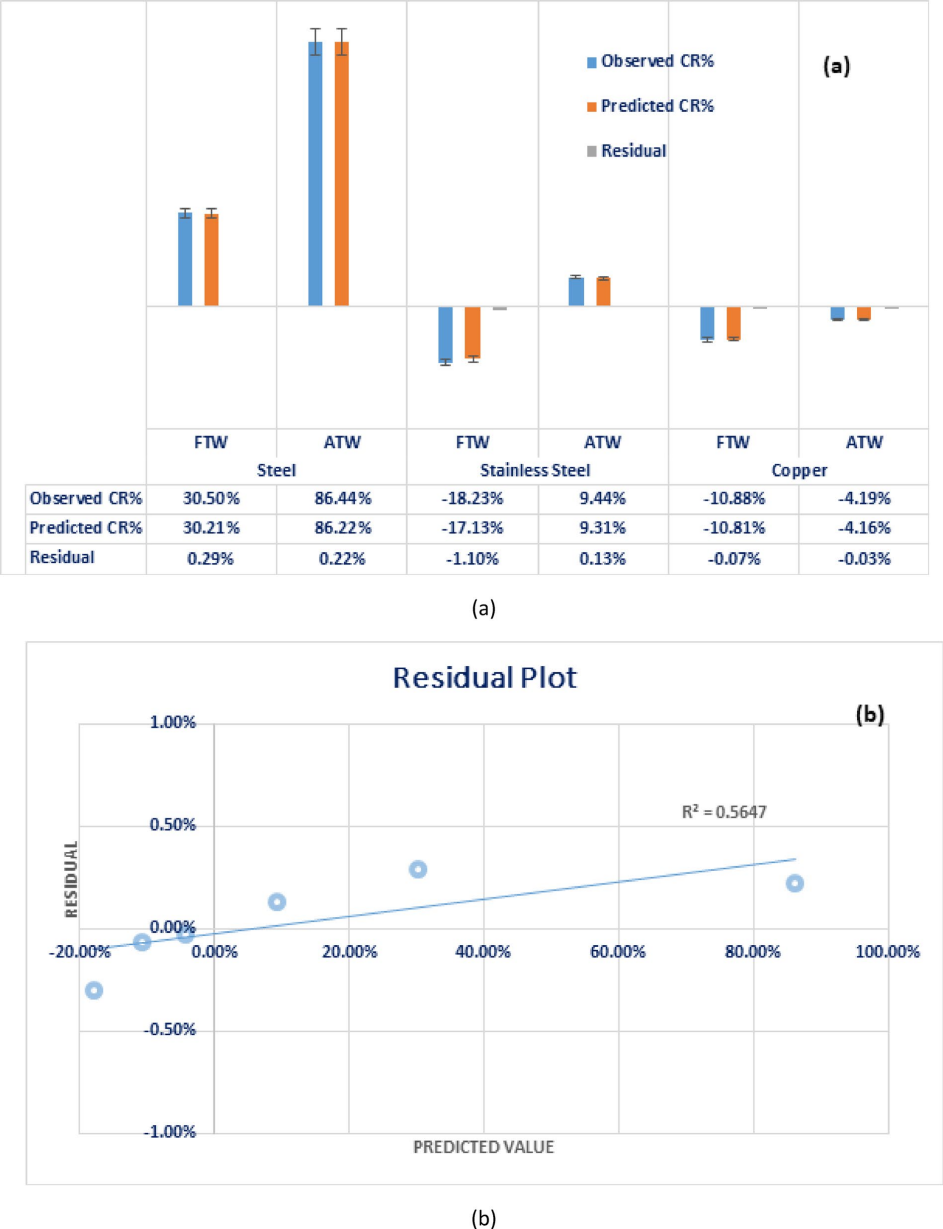
**(a)**Variation of observed data for steel corrosion *CR%* with predicted data (Dataset Size is limited; n = 9). **(b)** Residual plot (Predicted *CR%* Vs. Residual Value), the residual is randomly distributed along the predicted value.

The verification test was executed for the three models, illustrated in Eqs. 4,5 and 6 that can be used for the prediction of corrosion resistance of steel, stainless steel and copper respectively in various ceramic wastewater environments, the representation of observed and predicted *CR%* is illustrated in Fig. 8. That clarifies an excellent fit of three models with experimental data as well as small, obtained residual.

**Fig. 9:**
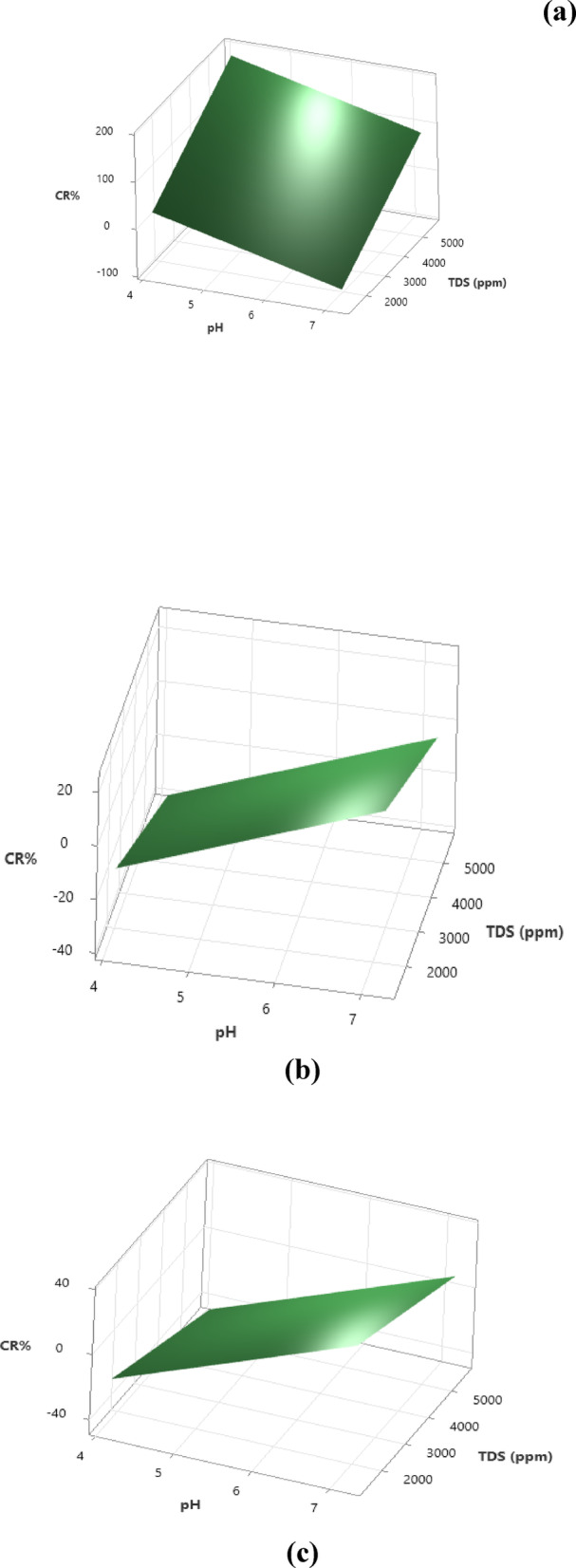
3-D Surface plot of **(a)** steel, **(b)** stainless steel, and **(c)** copper corrosion resistance, *CR%* Vs. *pH* and *TDS* of the ceramic wastewater treatment environment at constant *TP* content. *Model summary:* **(a)** *Dataset Size (n = 9) *CV* Score; (*R*^*2*^_pred_: 99.75%) at PRESS: 0.002833. **(b)** *Dataset Size (n = 9) *CV* Score; (*R*^*2*^_pred_: 99.81%) at PRESS: 0.0001357. and **(c)** *Dataset Size (n = 9) *CV* Score; (*R*^*2*^_pred_: 99.11%) at PRESS: 0.0001547 * Small and limited data set size.

Therefore, the development of these regression models provides feasible and effective ways to predict the corrosion resistance of mild steel, stainless steel, and copper in different ceramic wastewater treatment environments. Figure 9 illustrates the 3D surface plot for **(a) steel**, **(b) stainless-steel**, and **(c) copper** corrosion resistance as a function of *pH* and *TDS* of the different treatment environments.

**Fig. 10:**
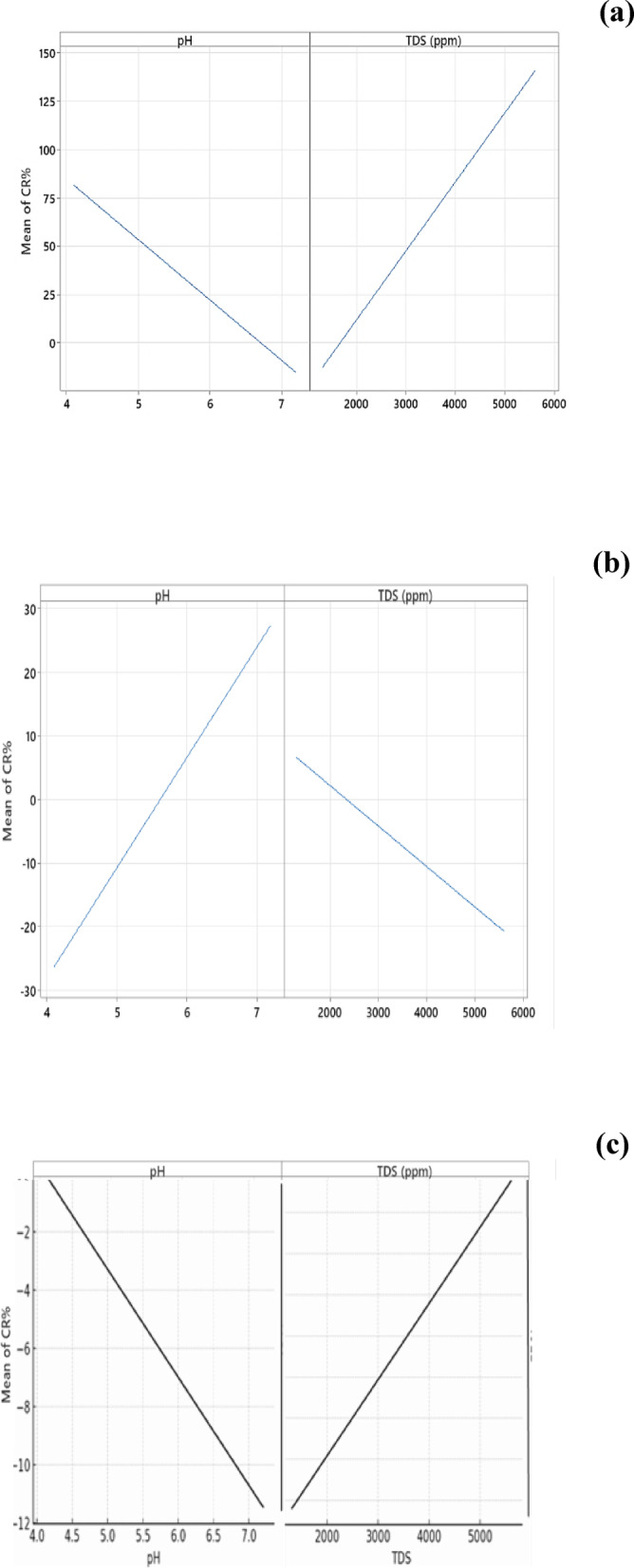
Main effects of **(a)** steel, **(b)** stainless-steel, and **(c)** copper, *CR%* Vs. *pH* and *TDS* (*ppm*) of the environment of different ceramic wastewater treatments *Model summary:* **(a)** *Dataset Size (n = 9) *CV* Score; (*R*^*2*^_pred_: 99.75%) at PRESS: 0.002833. **(b)** *Dataset Size (n = 9) *CV* Score; (*R*^*2*^_pred_: 99.81%) at PRESS: 0.0001357. and **(c)** *Dataset Size (n = 9) *CV* Score; (*R*^*2*^_pred_: 99.11%) at PRESS: 0.0001547 * Small and limited data set size.

Moreover, Fig. 10 represents the main effect of each predictor in the model, *pH* and *TDS* on the response “*CR%*” of (**a) steel**, **(b) stainless-steel** and **(c) copper** in different ceramic wastewater media, where the plots illustrate that the increasing of *pH* value of environment has a negative effect on steel and copper corrosion resistance meanwhile, has a positive effect on stainless-steel corrosion resistance. In contrast, increasing the *TDS* value has a positive effect on steel and copper corrosion resistance and a negative effect on stainless steel.

## Materials and methods

### Preparation of nano Zero Valent lron (nZVI)

Green nano-iron carbide was synthesized using the drop-by-drop method with black tea. In this study, the commercially sourced Kenyan black tea was chosen for its high and consistent polyphenol content. The phenolic compounds in the tea extract act as effective reducing agents for converting ferric ions (Fe^3^⁺) to zero-valent iron (Fe⁰), while simultaneously forming a protective organic coating on the nanoparticle surface to prevent oxidation and aggregation; this method is safer and environmentally benign than traditional chemical synthesis routes^67,68^.

**Fig. 11:**
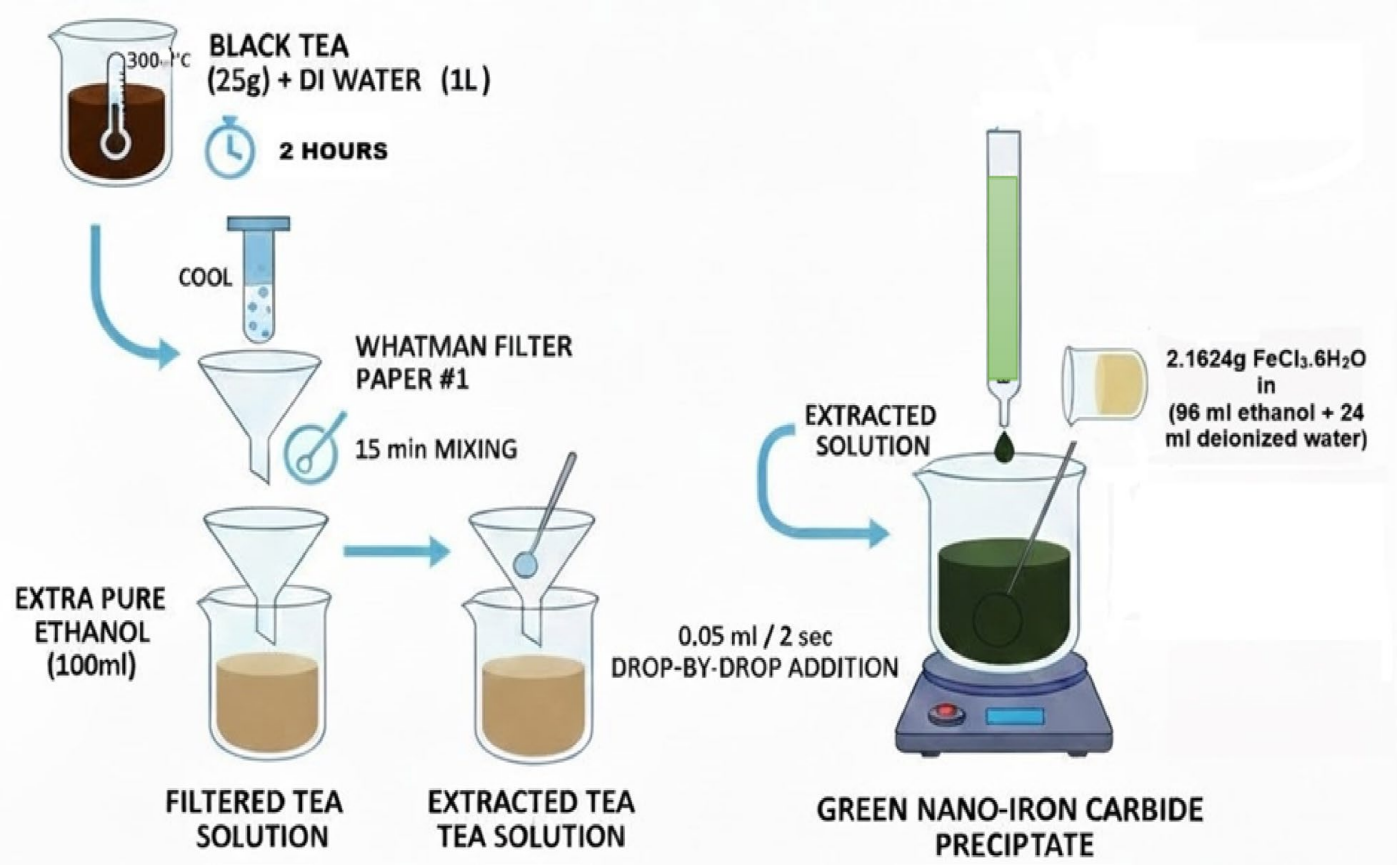
Schematic Diagram for nano Zero Valent Iron (nZVI) Preparation.

Approximately 25 g of black tea was combined in one liter of deionized water for two hours at 300 °C. After cooling, the extracted tea solution was filtered using Whatman filter paper #1. About 100 mL of extra pure ethanol solution was added to the infiltrating tea, mixed for 15 min and then filtered into the extracted tea solution. Approximately 2.1624 g *FeCl*_*3*_*.6H*_*2*_*O* was dissolved in a 4/1 (*v/v*) ethanol/water combination (96 mL ethanol + 24 mL deionized water). The extracted tea solution was put into a burette and added to the ferric chloride solution at a rate of 0.05 mL every 2 s while stirring with a magnetic stirrer at 150 rpm^69,70^, as illustrated in Fig. 11 (Schematic Diagram for nano Zero Valent Iron (nZVI) Preparation).

### Characterization of nano Zero Valent lron (nZVI):

The phase composition and crystallinity of the synthesized green nano zero-valent iron (nZVI) were investigated using X-ray diffraction (*XRD*). The diffraction pattern exhibited broad peaks at 2θ values of 44.58°, 64.99°, and 82.30° as shown in Supplementary Data (Fig.S1-a), corresponding to the (110), (200), and (211) planes of body-centered cubic (*BCC*) α-Fe, respectively. The peak broadening confirms the nanocrystalline nature of the *Fe*⁰ particles^71,72^.

Scanning electron microscopy (*SEM*) analysis revealed that the green nZVI particles possess irregular morphologies with rough and porous surfaces Supplementary Data (Fig.S1-b). The average particle size was approximately 45 nm. The observed porosity is expected to enhance surface area, promote mass transfer, and facilitate diffusion of organic species into the nanoparticle interior, thereby improving reactivity.

Particle size distribution analysis Supplementary Data (Fig.S1c) demonstrated that the synthesized powder is predominantly nanoscale, with 98.9% of particles within the nanometer range and approximately 95% exhibiting sizes below 70 nm. These results indicate a narrow size distribution and effective size control during green synthesis.

Energy-dispersive X-ray analysis (*EDAX*) confirmed the elemental composition of the selected nanoparticles Supplementary Data (Fig.S1d), showing iron as the dominant element along with oxygen and carbon, indicative of surface oxidation and a carbonaceous layer. Trace amounts of chloride and phosphorus were also detected, attributed to phytochemical constituents originating from the black tea extract. Collectively, these results confirm the successful synthesis of green nZVI with nanoscale dimensions and surface characteristics favorable for environmental remediation applications.

### Treatment methods

The study employed an advanced Fenton oxidation process followed by coagulation-precipitation and addition of green nano Zero Valent Iron nZVI for treating ceramic industry wastewater with a dosage of 100 ppm (0.1 g L^−1^). A lab-scale system was used to evaluate treatment efficiency, with optimization of key operating parameters (e.g., *H₂O₂/Fe*^*2*^⁺ ratio, *pH*, and reaction time) to determine optimal conditions. The final effluent quality was assessed for compliance with Egyptian Ministerial Resolution *No.92 of 2013*, ensuring adherence to regulatory standards for industrial wastewater discharge.

### Sample collection and characterization

Three distinct ceramic wastewater samples were collected from Ceramica Venezia: "Untreated Wastewater (UW)," which is ceramic wastewater before treatment; "Factory Treated Water (FTW)," which is wastewater treated by the factory’s initial treatment process; and "Advanced-treated water (ATW)," which is wastewater treated using the enhanced treatment process. The characteristics of these three samples are presented in Table 10.

**Table 10:** The physicochemical descriptions of three water samples: untreated wastewater (UW), factory-treated water (FTW), and advanced-treated water (ATW).

Sample	*pH*	*TDS (ppm)*	Conductivity (*µS/cm*)	T. Phosphorus (*mg/l*)
UW	5.1	1351	2390	1.70
FTW	4.1	1311	2320	0.30
ATW	7.2	5604	9310	0.52

### Corrosion rate measurements

Electrochemical tests were conducted using a three-electrode setup: first, a Saturated Calomel Electrode (*SCE*) as reference electrode, which was selected because it provides a constant, well-defined potential (0.244 V vs. SHE at 25 °C), which is essential for accurate measurements in aqueous environments. Second, a Graphite counter electrode, which was chosen due to its high electrical conductivity, chemical inertness in the chosen electrolyte, and high surface-to-volume ratio which enhances the sensitivity of the redox reaction. The working electrode was selected as mild steel, stainless steel, or copper, with a constant temperature of 30 ± 1 °C maintained. The metallic part of the working electrode was embedded in Teflon, allowing only one surface to be exposed to interact with the solution. This surface was then mechanically polished using a series of emery papers with varying grit sizes (100, 200, and 500 grits) and thoroughly washed with deionized water just before being placed in the electrochemical cell. Electrochemical impedance spectroscopy (EIS) and Potentiodynamic polarization (*PDP*) techniques were performed using the PARSTAT 2263 potentiostat as detailed in Supplementary Data (Section S1). To ensure reproducibility and statistical significance, all experiments were performed in triplicate (*n* = *3*) for each metal type and environmental condition.

### Statistical analysis

Corrosion resistance data were analyzed using ANOVA and Tukey’s post hoc test to determine significant differences between the three wastewater conditions. Statistical significance was set at *p* < *0.05*. The normality of the datasets was confirmed via the Ryan-Joiner test (*p* > *0.05*). All electrochemical measurements (*EIS* and *PDP*) were performed in triplicate (*n* = *3*) to ensure reproducibility. Data screening was performed by monitoring the stability of the Open Circuit Potential (*OCP*), scans that exhibit potential fluctuations more than 0.1 mV/s during the final 5 min of stabilization were excluded, and the experiment was repeated with a freshly prepared electrode surface.

### Regression modeling

Linear Regression Model was employed for the prediction of corrosion resistance of Steel, Stainless-steel, and Copper in different environments based on two continuous predictors, *pH* and *TDS* (*ppm*) of the medium. The model was validated using Leave-One-Out Cross-Validation due to the limited size of the data.

## Conclusion

The corrosivity effect of three distinct ceramic wastewater environments: "Untreated Wastewater (UW)," "Factory Treated Water (FTW)," which is wastewater that has undergone the factory’s initial treatment process; and "Advanced-treated water (ATW),” was investigated on three metals: mild steel, stainless steel, and copper.

Advanced-treated water (ATW), that include nZVI treatment, provides excellent corrosion resistance for steel, achieving an 86% reduction in steel corrosion rate compared to Untreated Wastewater (UW), which also exceeds the performance of the factory’s initial treatment process, “FTW” that achieves only 30% enhancement in corrosion resistance based on the results obtained from electrochemical impedance spectroscopy (*EIS*) and Potentiodynamic polarization measurements.

Additionally, the corrosion behavior of copper and stainless steel was examined, and the findings showed that the corrosion resistance of copper and stainless steel was somewhat enhanced in Advanced-treated water (ATW) as opposed to Untreated Wastewater (UW), while the corrosion resistance of both copper and stainless steel was found to be reduced in Factory Treated Water (FTW), although steel’s corrosion resistance was moderately improved, it is noteworthy also that neutral *pH* value of *ATW* enhanced helps all metals to resist corrosion than the acidic *pH* value of *FTW* .

Furthermore, the results of statistical validation supported the effectiveness of the advanced treatment process in Advanced-treated water (ATW) for wastewater recycling processes, improving equipment durability and ensuring sustainable water reuse. It also validated the results from Electrochemical Impedance Spectroscopy (*EIS*) and Potentiodynamic Polarization measurements, showing significant differences in corrosion behavior across different treatment stages.

Finally, Regression Modeling was employed to provide a model for the Prediction of the Effect of different ceramic wastewater environmental Parameters, *pH*, and *TDS* on the percentage corrosion resistance of Steel, stainless steel, and Copper, as well as demonstrating the feasible region and effective parameters for achieving the optimum corrosion resistance, While the *PLS* regression model demonstrated high predictive accuracy (*R*^*2*^_pred_ more than 99%), we acknowledge the limitation of a small dataset; therefore, these results serve as a preliminary proof-of-concept. Further validation with a larger, more diverse dataset is necessary to ensure broader generalizability.

### Future work

From the above discussion, the future research in this field should explore the following points:

1. Alternative corrosion protection for copper—investigating new aspects to be employed to minimize copper dissolution.
2. Long-term performance assessment—examining the stability and effectiveness of advanced treatment under prolonged operational conditions.
3. Integration with other industrial wastewaters—expanding the study to include wastewater from different industries to assess broader applicability.
4. Techno-economic feasibility—evaluating the cost-effectiveness and scalability of the enhanced treatment process for large-scale industrial implementation.

### Implications

The study’s conclusions have important ramifications for both industry and the environment:

- Industrial sustainability: using cutting-edge corrosion inhibitors can prolong the life of vital infrastructure, lowering maintenance costs and downtime.
- Environmental impact: efficient wastewater treatment reduces the discharge of hazardous materials into ecosystems, promoting environmentally friendly industrial practices.
- Water reuse potential: reusing treated wastewater for agricultural and industrial purposes lowers the demand for freshwater and advances the ideas of the circular economy.
- Policy and regulation compliance: strict environmental regulations and the adoption of improved treatment methods guarantee that industries continue to adhere to wastewater discharge standards.

## Supplementary Information

Below is the link to the electronic supplementary material.

Supplementary Material 1

## Data Availability

All data generated or analyzed are included within this article.
