# Supplementary material for: Sustainable treatment of ceramic manufacturing wastewater using combined advanced oxidation and coagulation/precipitation processes with green nano zero-valent iron: multi-metal corrosion monitoring
Source: Sci Rep. 2026 Mar 26;16:10491. doi: 10.1038/s41598-026-42824-1 (PMC13031382; doi:10.1038/s41598-026-42824-1)
Supplement: Supplementary file 1 — Supplementary Material 1 [file 41598_2026_42824_MOESM1_ESM.docx]

**Supplementary data**

**S1. Electrochemical Measurement Protocols**

To ensure the reproducibility of the corrosion kinetic data presented in the main manuscript, the following protocols for PDP and EIS were applied using the PARSTAT 2263 potentiostat.

**S1.1. Cell Configuration and Sample Preparation**

- **Three-Electrode Setup:** A standard electrochemical cell was used with a calomel (Ag/AgCl) reference electrode, a counter electrode (graphite), and the working electrode (WE) selected as mild steel, stainless steel, or copper., maintaining a constant temperature of 30 ± 1°C through pre-setting of water bath.
- **Inter-electrode distance**: a fixed distance of **20 mm** was maintained between the working and counter electrodes using a custom-designed cell.
- **Surface Preparation:** Prior to immersion, WE surface were ground using SiC papers with different grits up to 500 grits, rinsed with deionized water and dried.
- **Electrolyte:** 100 mL of untreated, factory-treated and advanced-treated wastewater samples from Ceramica Venezia.

**S1.2. Open Circuit Potential (OCP) Stabilization**

Before any dynamic measurements, the WE was immersed in the electrolyte for 30 minutes until a stable OCP was achieved “No external voltage was applied”.

**S1.3. Electrochemical Impedance Spectroscopy (EIS)**

EIS was performed first to avoid irreversible surface damage caused by high-voltage polarization.

- **Frequency Range:** 100 kHz to 10 mHz.
- **Equivalent Circuit Fitting:** Data were fitted using ZView 4

**S1.4. Potentiodynamic Polarization (PDP)**

Following EIS, PDP scans were conducted to determine kinetic parameters.

- **Scan Range:** -250 mV to +250 mV vs. SCE.
- **Scan Rate:** 0.167 mV/s.
- **Tafel Extrapolation:** Corrosion current density (i_corr_) was calculated by extrapolating the linear portions of the anodic and cathodic lines, ensuring the extrapolation started at least 50 mV away from E_corr_.


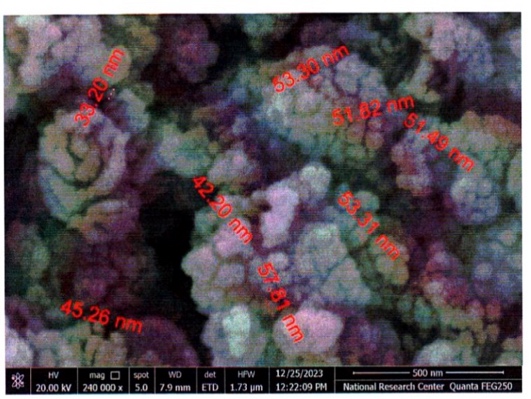

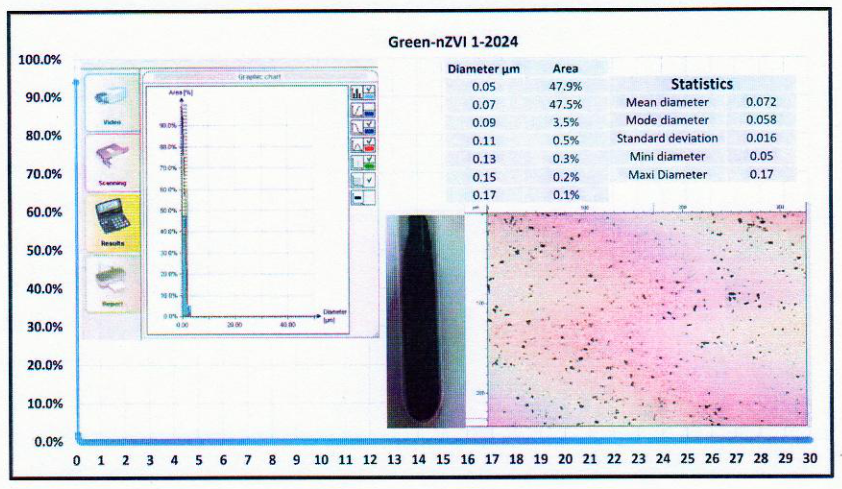

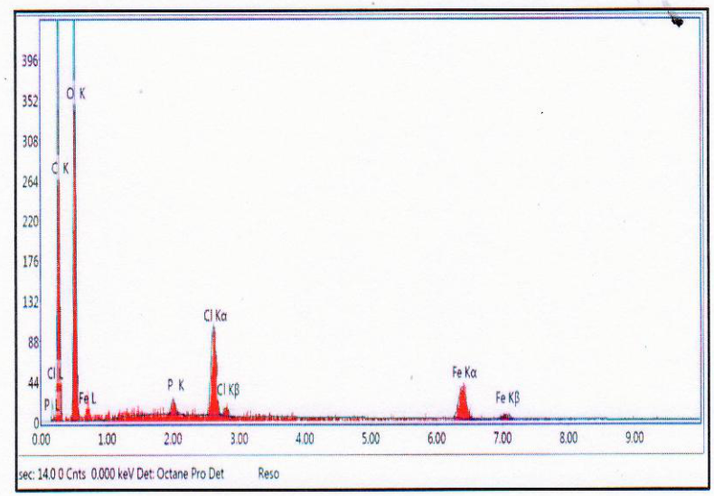

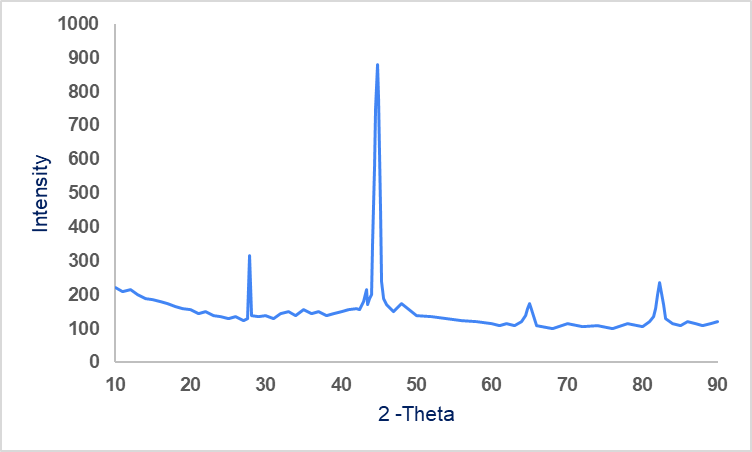


(a)

(b)

(d)

(c)

**Fig.S1**: Characterization of Green nZVI, (a) XRD spectrum, (b) SEM images, (c) Particle Size Distribution, (d) EDX spectrum
